# Plantar pressure thresholds as a strategy to prevent diabetic foot ulcers: A systematic review

**DOI:** 10.1016/j.heliyon.2024.e26161

**Published:** 2024-02-14

**Authors:** Pedro Castro-Martins, Arcelina Marques, Luís Coelho, Mário Vaz, José Torres Costa

**Affiliations:** a CIETI, ISEP, Polytechnic of Porto, Portugal; b Faculty of Engineering, University of Porto, Portugal; c Institute for Science and Innovation in Mechanical and Industrial Engineering, Porto, Portugal; d INESC-TEC, Centre for Robotics in Industry and Intelligent Systems, Porto, Portugal; e Faculty of Medicine, University of Porto, Portugal

**Keywords:** Diabetic foot, Plantar pressure, Plantar ulceration, Pressure threshold, Systematic review

## Abstract

**Background:**

The development of ulcers in the plantar region of the diabetic foot originates mainly from sites subjected to high pressure. The monitoring of these events using maximum allowable pressure thresholds is a fundamental procedure in the prevention of ulceration and its recurrence.

**Objective:**

The aim of this review was to identify data in the literature that reveal an objective threshold of plantar pressure in the diabetic foot, where pressure is classified as promoting ulceration. The aim is not to determine the best and only pressure threshold for ulceration, but rather to clarify the threshold values most used in clinical practice and research, also considering the devices used and possible applications for offloading plantar pressure.

**Design:**

A systematic review.

**Methods:**

The search was performed in three electronic databases, by the PRISMA methodology, for studies that used a pressure threshold to minimize the risk of ulceration in the diabetic foot. The selected studies were subjected to eligibility criteria.

**Results:**

Twenty-six studies were included in this review. Seven thresholds were identified, five of which are intended for the inside of the shoe: a threshold of average peak pressure of 200 kPa; 25 % and 40–80 % reduction from initial baseline pressure; 32–35 mm Hg for a capillary perfusion pressure; and a matrix of thresholds based on patient risk, shoe size and foot region. Two other thresholds are intended for the barefoot, 450 and 750 kPa. The threshold of 200 kPa of pressure inside the shoe is the most agreed upon among the studies. Regarding the prevention of ulceration and its recurrence, the efficacy of the proposed threshold matrix and the threshold of reducing baseline pressure by 40–80 % has not yet been evaluated, and the evidence for the remaining thresholds still needs further studies.

**Conclusions:**

Some heterogeneity was found in the studies, especially regarding the measurement systems used, the number of regions of interest and the number of steps to be considered for the threshold. Even so, this review reveals the way forward to obtain a threshold indicative of an effective steppingstone in the prevention of diabetic foot ulcer.

## Teaser

Tweetable abstract The development of ulcers in the plantar region of the diabetic foot originates mainly from sites subjected to high pressure. The monitoring of these events using maximum allowable pressure thresholds is a fundamental procedure in the prevention of ulceration and its recurrence. A systematic review was conducted with the purpose of answering the following research question: Which measurable pressure thresholds are most relevant to identify the risk of plantar ulceration in diabetic patients? The threshold of 200 kPa inside the shoe is the most agreed, although some heterogeneity was found in the studies, especially regarding the measurement systems used, the number of regions of interest and the number of steps to be considered for the threshold. Even so, this review reveals the way forward to obtain a threshold indicative of an effective steppingstone in the prevention of diabetic foot ulcer.

## 1 Introduction

Diabetes is an incurable chronic disease with an exponential growth trend worldwide. According to the 2021 report of the International Diabetes Federation [1], about 537 million adults are living with diabetes, advancing a projected increase to 643 million people with diabetes by 2030 and 784 million by 2045. In 2021 this disease was responsible for 6.7 million deaths and caused health expenditures of 862 billion euros [1].

The diabetic patient has an increased susceptibility to develop diabetic foot, a complication that translates mainly into ulceration of some regions of the feet. This is a frequent consequence due to comorbidities such as diabetic neuropathy, peripheral vascular disease, foot deformities, and pressure injuries caused by the patient's footwear during gait [[2], [3], [4], [5], [6], [7]]. These comorbidities are at the genesis of the loss of protective cutaneous sensitivity, which interrupts the patient's protective cutaneous feedback mechanism by drastically reducing pain sensitivity [8]. This condition makes these patients more susceptible to trauma that can precipitate an injury and easily develop into an ulcer [[9], [10], [11], [12]].

Because of these complications it is expected that about 34 % of people with diabetes will develop at least one foot pressure ulcer during their working life. The level of recurrence of ulcers after healing is 40 % in the first year and increases to about 60 % in three years [13]. These lesions are highly associated with infection and limb amputation [14].

The development of diabetic foot ulcers originates mainly in places of the plantar region subject to greater pressure, such as the metatarsal heads, toes, and heel. Although less frequent, the dorsal region can also be affected by ulcers, mainly on the bony prominences of the toes [15]. For this reason, the mapping of hyperpressure sites is used to guide the manufacture of custom footwear, orthotics, and insoles adapted to each patient with the goal of reducing these pressures at a particular location on the foot. This procedure, and the use of this equipment to relieve the pressures, during a treatment phase or permanently, sometimes conditions the natural locomotion of the user [16,17].

Supervision, reduction and/or redistribution of plantar pressures are essential actions to avoid injury or accelerate ulcer healing and prevent recurrence [18,19]. For this purpose, active monitoring of these pressures, through technologies integrated into the footwear, begins to replace the traditional procedure of measuring platforms that are used only in clinical settings and with the barefoot. Sensors integrated into the footwear itself, in removable insoles or socks, are some examples [[20], [21], [22], [23]]. In this sense, these monitoring systems need to know what the admissible pressure threshold for the generality of these patients, where, once exceeded, an alert is triggered by the occurrence of hyperpressure [24,25].

Some studies, including systematic reviews of the literature, address the possible effectiveness of insoles and other systems in measuring plantar pressures and gait analysis, highlighting efforts to identify pressure peaks and define preventive thresholds for diabetic foot ulcers. Despite the reported advances, there is still a need for additional studies to establish a reliable threshold [16,17,21]. Considering this, what is novel about our systematic review is that it aims to aggregate scattered information about the most relevant plantar pressure thresholds in practice and clinical research for treatment and to avoid possible ulcerations when pressure measurement systems are used as prevention. With this information we have devised a set of plantar pressure ranges and thresholds which we have associated with specific applications and contexts, encompassing both prevention and therapeutic approaches within the scope of the diabetic foot. As far goes the authors knowledge this type of revision and information gathering was not reported in the current scientific literature.

In this systematic review, the focus was to systematically review the existing literature covering the definition of an objective threshold of plantar pressure in the diabetic foot where pressure is classified as an ulceration promoter. Within this objective we want to understand in what conditions this threshold is applied to promote an effective reduction of plantar pressure, preventing, and helping to treat ulceration or relapse, encompassing all the stages involved in diabetic foot management. This systematic literature review does not aim to determine the best and only pressure threshold for ulceration, but rather, to aggregate information with the aim of clarifying the threshold values most used in clinical practice and research and the related contexts while also considering the used devices and possible applications for offloading plantar pressure in diabetic foot.

## 2 Materials and methods

This systematic review was conducted and structured according to the guidelines proposed by PRISMA methodology – Preferred Reporting Items for Systematic Reviews and Meta-Analyses [26]. In this literature review, we chose to carry out only a systematic review according to the PRISMA methodology, without providing a meta-analysis.

### 2.1 Research strategy

**Fig. 1:**
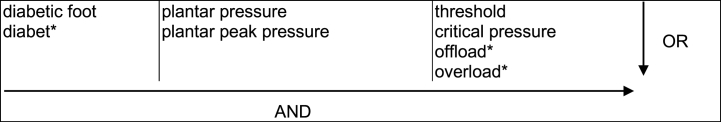
Keywords used in the search performed in the electronic databases.

A literature search was conducted in three scientific repositories – Scopus, PubMed, and Web of Science – in December 2021 (updated in April 2023). These electronic databases were chosen because they are recognized by the scientific community and because their content is compatible with the areas under study. They were screened using keywords (Fig. 1) properly selected to restrict the data obtained to the subject of study and maximize the article identification process. The common search query for all electronic databases was: (diabetic foot OR diabet*) AND (plantar pressure OR plantar peak pressure) AND (threshold OR critical pressure OR offload* OR overload*). This query was the most consistent for all electronic databases consulted and the one that returned the best results. In addition, articles identified in the citations of the respective authors during the screening process were included, which were also subject to the eligibility criteria.

### 2.2 Selection and eligibility criteria

The information selection method considered published articles without any additional restrictions (date, document type, source type and language) to broaden the data mining. In a first approach, duplicate records were removed. Next, the titles and abstracts of the articles were analyzed to classify them as being on-topic, and those that did not fit the topic were rejected. Articles that addressed the topic of assessment and quantitative reduction of plantar pressure through a certain threshold, intended for people with diabetic foot or similar applications, were classified as on-topic. In the next step, the full text of the considered articles was reviewed and eligibility criteria were applied, selecting those articles that cumulatively met the following requirements: i) studies directed to diabetic patients with diabetic foot previously diagnosed by their attending physician, or classified with high probability of developing ulcers or complications by the association of comorbidities such as neuropathy (classification according to the IWGDF guidelines – International Working Group on the Diabetic Foot [3,27]); ii) focusing on the measurement of plantar pressure and using – or obtaining in their results – a quantitative threshold for plantar pressure; iii) and directed towards reducing plantar pressure and/or studying its effects by applying a given plantar pressure threshold; iv) other similar applications aimed at controlling the effects of plantar pressure on the diabetic foot according to a quantitative pressure threshold.

The study aimed to evaluate the acceptability of plantar pressure threshold values in different scenarios, using a quantitative analysis as a reference. However, to avoid bias, case reports were excluded from the review due to their purely qualitative characteristics. Furthermore, a minimum cohort size of 10 individuals was established for the intervention, with or without a control sample. Considering the large population of patients with diabetic foot, for us this minimum intervention sample was defined from the beginning as a minimum acceptable size. Randomized controlled trials (RCT), cohort and case-control studies were included. The article selection process was carried out by two independent authors, PCM and LC, with divergences regarding the inclusion or not of a given article were resolved by a third author, AM, to guarantee objectivity in the selection of articles.

### 2.3 Data extraction

Once the selection process of eligible articles was completed, the following data were collected: i) identification of the authors, year of publication and title; ii) research topic and objectives; iii) methodology applied; iv) results obtained and conclusions. The data extracted were useful to answer a set of questions and to perform an objective synthesis of the information, relating the different approaches and criteria to establish an objective plantar pressure threshold. Data extraction was performed by PCM and was subsequently reviewed and validated by LC. Additionally, and carried out by the same authors, the quality of studies included in this systematic review was assessed using the Newcastle-Ottawa Quality Assessment Scale [28] for cohort and case-control studies. Studies with Newcastle-Ottawa scores of 0–3, 4–6 and 7–9 were considered as poor, fair, and good quality, respectively. For RCT studies the Jadad Scale [29] was used, where a score of 0–2 points is considered poor quality and a score of 3–5 points is considered good quality.

## 3 Results

**Fig. 2:**
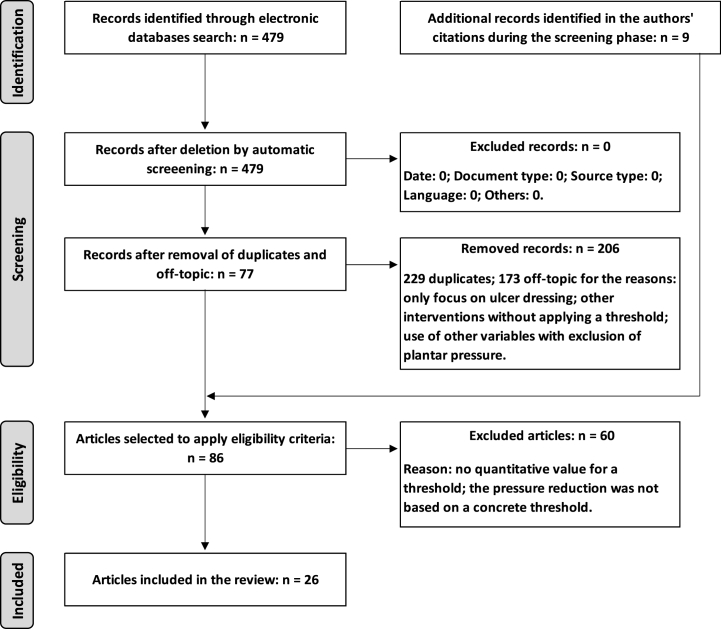
PRISMA methodology flow diagram for the article selection process.

The search and selection process, illustrated in Fig. 2, initially identified a total of 479 records (Scopus: 172; PubMed: 135; Web of Science: 172), spanning a time window from 1989 to 2022. Of these, 229 duplicates and 173 classifieds as off-topic were excluded. After this screening, 86 articles remained (with inclusion of 9 articles identified in the authors' citations) for application of the eligibility criteria. In the end, 26 studies were effectively included in this review.

### 3.1 Description of included studies

The information collected revealed the plantar pressure thresholds currently used to promote pressure reduction and aid in the treatment and prevention of diabetic foot ulceration. Most studies were conducted from 2008 to 2022, only one study [30] is older, conducted in 1998. Twenty-two studies [25,[31], [32], [33], [34], [35], [36], [37], [38], [39], [40], [41], [42], [43], [44], [45], [46], [47], [48], [49], [50], [51]] focus on reducing plantar pressure by applying pressure thresholds on the foot solely inside the shoe. One study [30] presents only pressure thresholds for the barefoot foot with emphasis on identifying regions of interest in the foot or classifying risk groups. Three other studies [[52], [53], [54]] expose a hybrid methodology, in addition to making pressure measurements inside the shoe, they also indicate thresholds for barefoot pressure. A summary of the information from the studies included in this systematic review can be seen in Table 1.

**Table 1:** Summary of information regarding the twenty-six studies included in the review.

Author, year	Purpose	Participants: n (M/F); age ± SD		Duration	Regions of Interest	Measuring System (method)	Pressure threshold and criteria applied
		Intervention	Control				
Maharana, 2022 [51]	Design and customization of shoe arches for dynamic offloading.	71 (−/−); 55 ± 10.9	29 (−/−); 56.1 ± 8.7	–	10 subregions distributed through the plantar region.	Pedar-X (insole)	200 kPa. Pressure value < 200 kPa used to consider effective pressure reduction and avoid injuries from prolonged high plantar pressure.
**Muir**, 2022 [50]	Test the effect of standard and 3D-printed diabetic insoles on reducing plantar pressure.	12 (8/4); 63.8 ± 9.2	–	–	In the place of the plantar region where the high-pressure level is presented.	Pedar-X (insole)	200 kPa. A pressure value < 200 kPa is used to consider an effective pressure reduction and 200 kPa used as the maximum threshold for injury risk to occur.
**Cao**, 2021 [31]	Reduce plantar pressure through appropriate footwear.	19 (10/9); 65.7 ± 3.4 17 (9/8); 65.2 ± 6.8	20 (11/9); 65.2 ± 5.4	1 session	7 sub-regions distributed over the plantar region.	Medilogic 5.8.1 (insole)	200 kPa. All measurements with pressures above 200 kPa are considered as hyperpressure.
**Abbott**, 2019 [32]	Reducing plantar pressure with the aid of a biofeedback monitoring system.	32 (28/4); 59.1 ± 8.5	26 (23/3); 67.1 ± 9.6	18 months	8 sub-regions distributed over the plantar region.	SurroSense RX (insole)	35 mm Hg (capillary perfusion pressure as an indirect measure of increase plantar pressure). Suggested ulceration risk for all pressures ≥35 mm Hg. If in the last 15 min of measurement 95 % or more readings are ≥35 mm Hg an alert is issued for the patient to correct gait and relieve pressure at the identified local.
**Martinez-Santos**, 2019 [33]	Reducing plantar pressure through a customized insole.	60 (40/20); 67.0 ± ---	–	1 session	5 sub-regions distributed in the plantar forefoot.	Pedar-X (insole)	200 kPa. A pressure value < 200 kPa is used to consider an effective pressure reduction, in 20 steps.
**Parker**, 2019 [34]	Identify risk regions and reduce plantar pressure through orthoses.	57 (50/7); 61.4 ± 10.0	–	6 months	3 sub-regions distributed in the plantar forefoot.	Pedar (insole)	200 kPa. The risk regions were identified and classified considering plantar pressure >200 kPa in 30 steps/foot.

Author, year	Purpose	Participants: n (M/F); age ± SD		Duration	Regions of Interest	Measuring System (method)	Pressure threshold and criteria applied
		Intervention	Control				
**Searle**, 2018 [52]	Evaluate the equinus foot and investigate any clinical effects on plantar pressure.	104 (54/50); 73.3 ± 5.6	40 (16/24); 28.8 ± 7.9	1 session	5 sub-regions distributed throughout the plantar region, with greater focus on the forefoot.	Pedar-X (insole) Tekscan HR Mat (barefoot)	In-shoe: 200 kPa. Pressures ≥200 kPa are classified as hyperpressures, in 12 steps/feet. Barefoot: 700 kPa. Pressures >700 kPa barefoot serve to identify groups with current or recently healed ulcers.
**Preece**, 2017 [35]	Evaluate optimal footwear design and appropriate pressure reduction.	102 (52/50); 57.0 ± 9.0	66 (36/30); 56.0 ± 8.0	1 session	3 sub-regions distributed in the plantar forefoot.	Pedar-X (insole)	200 kPa. Footwear intended to prevent first ulceration must ensure a pressure <200 kPa at 25 steps/foot.
**Telfer**, 2017 [53]	Comparing performance in pressure relief through optimized insoles.	18 (5/13); 64.4 ± 9.2	–	1 session	3 sub-regions distributed in the plantar forefoot.	Pedar-X (insole) Emed platform (barefoot)	In-shoe: 200 kPa. 200 kPa threshold used to evaluate insoles and customize them, in 12 steps/foot. Barefoot: 450 kPa. Barefoot pressure threshold of 450 kPa used to select regions of interest.
**Van**, 2017 [36]	Evaluating the biofeedback method for reducing plantar pressure.	11 (−/−); ---	–	Up to 6 weeks	Plantar region.	FeetMe One (insole)	40–80 % reduction. 40–80 % reduction in peak pressures for 70 % of the total steps during walking, every 20 steps.
**Arts**, 2015 [37]	Reduce plantar pressure by modifying footwear.	85 (70/15); 62.6 ± 10.2	–	15 months	8 sub-regions distributed across the forefoot and midfoot.	Pedar-X (insole)	200 kPa. Footwear modified in the area where an average peak pressure ≥200 kPa occurs, in 12 steps/foot.
**Chapman**, 2014 [38]	Reduce plantar pressure through balance shoes and orthotics.	87 (−/−); ---	–	1 session	Plantar region.	---(in-shoe)	200 kPa. Maximum threshold of 200 kPa used to identify an effective pressure reduction.

Author, year	Purpose	Participants: n (M/F); age ± SD		Duration	Regions of Interest	Measuring System (method)	Pressure threshold and criteria applied
		Intervention	Control				
**Waaijman**, 2014 [39]	Identify risk factors for ulcer recurrence.	171 (141/30); 63.3 ± 10.1	–	18 months	10 sub-regions distributed over the plantar region.	Pedar-X (insole)	200 kPa. Pressure >200 kPa used to classify participants into groups above the ulceration risk threshold, in 12 steps/foot.
**Bus**, 2013 [40]	To evaluate the effect of wearing customized shoes on pressure reduction.	171 (140/31); 63.3 ± 10.2	–	18 months	Midfoot and forefoot.	Pedar-X (insole)	25 % reduction or 200 kPa. Shoe modifications until one of the identified thresholds is reached, whichever occurs first, in 15 steps.
**Rodriguez**, 2013 [41]	Evaluate biofeedback corrected walking strategy to reduce plantar pressure.	21 (12/9); 57.8 ± 2.9	–	10 days	11 sub-regions distributed over the plantar region.	Pedar-X (insole)	40–80 % reduction. The new walking strategy should reduce the base pressure in the shoe by 40–80% for 7 out of 10 steps.
**Ferber**, 2013 [42]	Compare instrumented insoles to determine the correlation between simultaneous measurements based on a given threshold.	10 (7/3); 27.9 ± 6.6	–	1 session	8 sub-regions distributed over the plantar region.	SurroSense RX (insole) Pedar-X (insole)	32 mm Hg (capillary perfusion pressure as an indirect measure of increase plantar pressure). Suggested ulceration risk for all pressures ≥32 mm Hg.
**Giacomozzi**, 2013 [43]	Validate new protocol to apply pressure thresholds customized according to the patient's risk level.	11 (−/−); 70.2 ± 6.7	20 (−/−); 67.0 ± 7.9	4 months	4 sub-regions distributed over the plantar region.	Pedar-X (insole)	Pressure matrix (see Table 3). Different thresholds for four regions of the foot and stratified by risk level and shoe size, for every 20 steps/foot.
**Lin**, 2013 [44]	Evaluating the effect of customized insoles on pressure reduction.	26 (10/16); 68.0 ± 9.0	–	1 session	5 sub-regions distributed in the plantar forefoot.	Pedar-X (insole)	200 kPa. The ulceration risk site is the forefoot region where the highest average peak pressure value is > 200 kPa in 30 steps.
**Arts**, 2012 [45]	Evaluate pressure reduction through customized footwear.	171 (140/31); 62.8 ± 10.2	–	1 session	10 sub-regions distributed over the plantar region.	Pedar-X (insole)	200 kPa. Pressure reduction is successful if it is at levels <200 kPa, every 12 steps/foot.

Author, year	Purpose	Participants: n (M/F); age ± SD		Duration	Regions of Interest	Measuring System (method)	Pressure threshold and criteria applied
		Intervention	Control				
**Waaijman**, 2012 [46]	To evaluate the effect of wearing customized shoes on pressure reduction.	117 (−/−); 63.3 ± 10.1	32 (−/−); ---	12 months	10 sub-regions distributed over the plantar region.	Pedar-X (insole)	25 % reduction or 200 kPa. Reduction of the peak pressure by 25 % or an absolute level <200 kPa Shoe modification based on the anterior location of the ulcer and/or the two sites with pressure >200 kPa, in 20 steps.
**Bus**, **2011** [47]	To evaluate the effect of wearing customized shoes on pressure reduction.	23 (17/6); 59.1 ± 12.6	–	1 session with multiple trials	10 sub-regions distributed over the plantar region.	Pedar-X (insole)	25 % reduction or 200 kPa. Shoe personalization is successful if the pressure reduces by at least 25 % of the average peak value at that location or is < 200 kPa, in 15 steps.
**Waaijman**, 2011 [48]	To evaluate the effect of wearing customized shoes on pressure reduction.	58 (−/−); 62.6 ± 10.3	–	–	Plantar region.	Pedar-X (insole)	25 % reduction or 200 kPa. Shoe modifications until one of the thresholds is reached.
**Pataky**, 2010 [25]	Evaluate biofeedback-corrected walking strategy to reduce plantar pressure.	13 (6/7); 60.8 ± 12.3	–	Up to 10 days	Previously classified risk area in the plantar region.	Pedar (insole)	40–80 % reduction. The new walking strategy should reduce the base pressure in the shoe by 40–80 % for 7 out of 10 steps.
**Owings**, 2009 [49]	Measuring plantar pressures and providing information for a given threshold of ulceration prevention.	49 (38/11); 62.9 ± 10.3	–	1 session	3 sub-regions distributed in the plantar forefoot.	Pedar (insole)	200 kPa. Set a target pressure of 200 kPa to prevent ulcer recurrence, in 30 steps.
**Owings**, 2008 [54]	Evaluating the effect of customized insoles on pressure reduction.	20 (10/10); 63.7 ± 10.7	–	1 session	4 sub-regions distributed over the medial and forefoot.	Pedar-X (insole) Emed-D Platform (barefoot)	In-shoe: no threshold or criteria, just to measure pressure. Barefoot: 450 kPa. Barefoot pressure threshold of 450 kPa used to select regions of interest, in the first step.
**Armstrong**, 1998 [30]	Identify a threshold of peak forefoot plantar pressure that has a sensitivity and specificity for tracking ulceration.	70 (52/18); 52.3 ± 10.3	149 (50/99); 51.8 ± 10.4	1 session	Plantar region.	Emed platform (barefoot)	Barefoot: 700 kPa. Pressures >700 kPa barefoot serve to identify groups at higher risk of ulceration, for every 3 step/foot.

Regarding the quality assessment of the studies included in this systematic review, according to the Newcastle-Ottawa Quality Assessment Scale for cohort and case-control studies, 7 studies received high score (7–9, representing good quality and low risk of bias) and 16 studies received a moderate score (4–6, fair quality with the possibility of presenting some risk of bias). For the RCT studies, using the Jadad Scale, all 3 studies were classified as good quality, which implies a low risk of bias. The quality assessment scores can be consulted in Table 2.

**Table 2:**
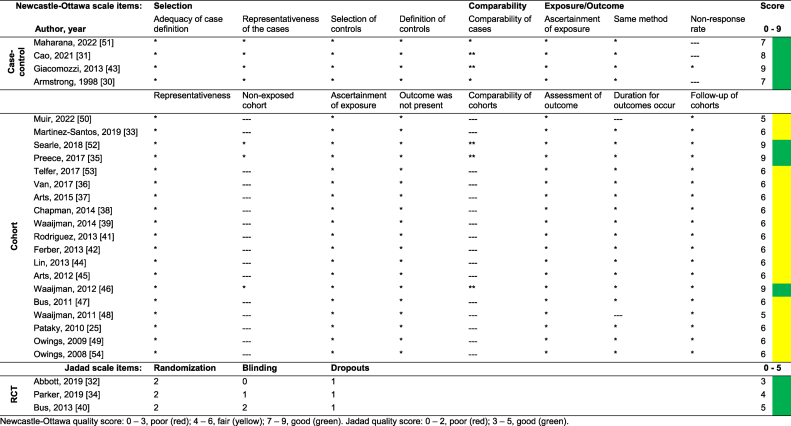
Scores of the Newcastle-Ottawa Quality Assessment Scale (cohort and case-control) and Jadad Scale (RCT) for the included studies.

**Table 3:** Matrix of pressure thresholds for the plantar region stratified by risk level and European (EU) shoe size [43,55]. Risk level: (0) low - no neuropathy, no history of ulceration or foot deformity; (1) medium - with neuropathy, but no history of ulceration or foot deformity; (2) high - with neuropathy and foot deformity, but no history of ulceration; (3) very high - with neuropathy, foot deformation, and history of ulceration.

Risk Level	EU shoe size	Pressure threshold (kPa)			
		Rearfoot	Midfoot	Forefoot	Toes
0–1	36/37	230	100	270	160
	38/39	200	90	240	150
	40/41	180	80	220	130
	42/43	160	70	200	120
	44/45	150	70	180	110
2	36/37	210	60	240	140
	38/39	180	60	220	130
	40/41	160	60	200	120
	42/43	140	60	180	110
	44/45	130	60	160	100
3	36/37	180	60	220	130
	38/39	160	60	190	120
	40/41	140	60	180	100
	42/43	130	60	160	100
	44/45	120	60	140	90

***Geographical distribution*.** The selected studies are mainly concentrated in Europe (75 %), and to a lesser extent in North America (12.5 %), Asia (8.3 %) and Oceania (4.2 %).

***Participants and demographics.*** The studies included groups of participants for intervention and control that, in total, ranged from 10 [42] to 219 [30] people. The target population mainly comprised diabetic patients for the intervention groups, but also healthy people for some control groups. The intervention groups are heterogeneous from study to study, consisting of diabetics with/without neuropathy, with/without history of ulceration, and with/without foot deformity. The age of the participants, when provided, including healthy people for the control groups, is between 27.9 ± 6.6 [42] and 73.3 ± 5.6 [52] years. The gender ratio, when provided, showed male participation ranging from 5 [53] to 141 [39] persons, and female from 3 [42] to 117 [30].

***Purpose of the studies.*** The studies' approaches were always expected to reduce plantar pressure based on a certain threshold. Some studies proposed to assess the ability to reduce plantar pressure by: wearing custom footwear [31,35,37,38,40,[45], [46], [47], [48],^52^]; custom insoles [33,44,50,51,53,54]; orthotics [34,38]; and through a biofeedback alert for the user to adopt a gait pattern capable of relieving pressure in the risk region indicated by the system [25,32,36,41]. One study compared the ability of two insole systems for pressure measurement to detect a low capillary perfusion pressure, as an indirect measure of plantar pressure threshold [42]. Other studies were based on identifying ulceration risk factors and regions associated with pressure level and suggesting appropriate thresholds to prevent ulceration [30,39,43,49,52].

### 3.2 Pressure measurement systems

**Fig. 3:**
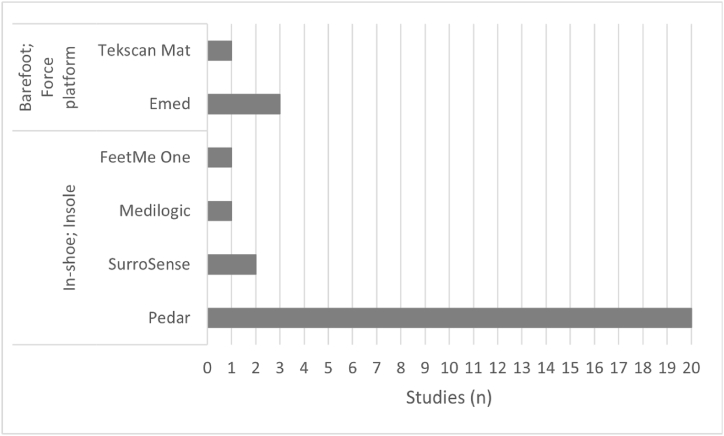
Distribution of studies according to the plantar pressure measurement systems used, grouped by in-shoe and barefoot scenarios. Studies that used more than one measurement system were counted in the respective system groups.

Different systems of plantar pressure measurement were identified. For measurements inside the shoe, instrumented insoles with sensors distributed across the plantar region have been used [25,[31], [32], [33], [34], [35], [36], [37],[39], [40], [41], [42], [43], [44], [45], [46], [47], [48], [49],[52], [53], [54]]. Barefoot measurements were performed using pressure platforms [30,[52], [53], [54]]. Fig. 3 presents a distribution between the systems used to measure plantar pressure and the number of studies that used them. This provides an overview of the most used systems for plantar pressure assessment and the various applications for diabetic foot management.

***Instrumented insoles.*** The Pedar system, based on an instrumented insole for insertion inside the shoe, has been the most widely used and considered the gold standard system for this type of application [25,[33], [34], [35],^37^,[39], [40], [41],[43], [44], [45], [46], [47], [48], [49],[52], [53], [54]]. It consists of a thin structure to couple inside the shoe and contains 99 sensors in the plantar region that can be subdivided into regions of interest. Other similar systems have also been used, such as the Medilogic insoles [31], SurroSense [32,42] and FeetMe [36]. The SurroSense (with 8 sensors) and FeetMe insoles, in addition to measuring the reduction of maximum peak pressure, were also used in a gait adaptation strategy through a biofeedback hyperpressure alert, indicating to the user the region at risk for him to compensate his gait pattern and relieve the pressure in that area of the foot [32,36].

***Pressure platforms.*** For the barefoot measurements Tekscan HR Mat pressure measurement platforms were used [52] and Emed [30,53,54]. They are usually only used in a controlled clinical or laboratory setting. The procedure consisted in locomotion of the patient with bare feet on the platform surface and, at this moment, the pressures exerted by the plantar region of the feet on the platform were recorded, also enabling the identification of potential regions of study interest [30,[52], [53], [54]].

### 3.3 Regions of interest

The plantar region of the feet was the focus of the studies, however, this was divided into 3 areas: rearfoot, midfoot, and forefoot. Within these, the authors defined regions of interest that ranged from 3 [34,35,49,53] up to 11 [41] subregions, distributed in one of these areas or across the entire plantar region. These subregions comprised the heel, the lateral and medial midfoot, the heads of the five metatarsals, and the toes. These are sites where the highest pressure events occur or, the previous sites of ulceration to prevent their recurrence [[45], [46], [47], [48]].

### 3.4 Pressure thresholds inside the shoe

**Fig. 4:**
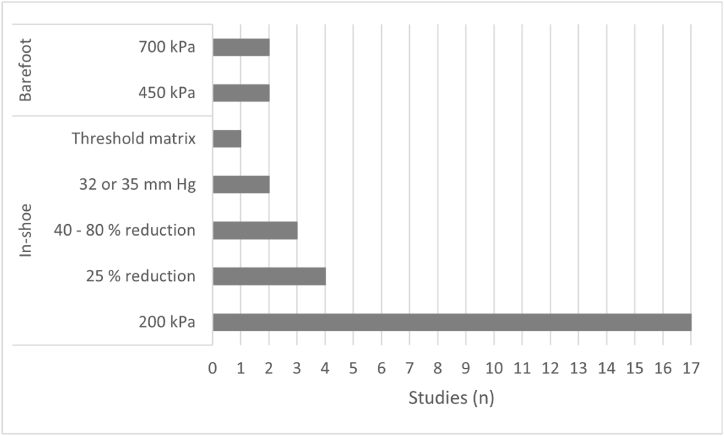
Distribution of studies according to pressure thresholds, grouped by in-shoe and barefoot scenarios. Studies that used more than one threshold were counted in the respective threshold groups.

Five different thresholds for managing plantar foot pressure inside the shoe have been identified. The authors use them as target thresholds for an effective and successful pressure reduction to avoid injury and ulceration. Fig. 4 presents a distribution between the thresholds applied in the management of plantar pressure and the number of studies that used them, both for applications inside shoes and with the barefoot. This provides an overview of the most used thresholds for plantar pressure management and the various research applications on ways to offloading plantar pressure in the diabetic foot.

***Threshold of* 200 kPa*.*** Of the thresholds identified, the threshold of 200 kPa mean peak pressure was the most widely used and cut across the majority (about 67 %) of studies. This threshold is applied to the set of measurements taken during a walk for every 12 steps per foot [37,39,45,52,53] up to 30 steps per foot [34] or, from 15 [40,47] up to 30 common steps [44,49]. The authors of the studies used the 200 kPa ceiling of plantar pressure to: monitor the previous ulcer site and determine risk factors (pressure) for recurrence [39,49]; identify regions of interest where hyperpressures occur [34]; customize or evaluate footwear [31,35,37,40,[45], [46], [47], [48]], insoles [33,44,53] and orthotics [34,38] with the aim of promoting foot pressure reduction; and to evaluate the limitations imposed by the equinus foot condition in diabetic patients and its repercussion on the increase of plantar pressure [52].

***Pressure reduction by 25 %.*** In addition to the established threshold of 200 kPa, four studies also propose a second alternative, suggesting a 25 % reduction in the baseline pressure characteristic of a given patient [40,[46], [47], [48]]. This alternative arises because it was considered to have clinical relevance due to some participants having pressures below the threshold of 200 kPa but were still at risk of developing or had developed an ulcer. Others, with pressures above the threshold did not develop an ulcer [47,48]. This may have been a factor in considering the threshold of 200 kPa or an effective reduction by 25 % of baseline pressure, whichever occurs first [40].

***Pressure reduction by 40 - 80 %.*** A more ambitious threshold reduction is proposed by three studies and consists of lowering the initial baseline pressure by 40–80 % [25,36,41]. The proposed reduction is based, after a prior alert by biofeedback, on learning and adapting the gait pattern with the aim of relieving pressure in a certain region of the foot, properly signaled in this alert. This reduction interval is monitored and calculated during the gait for every 7 of 10 steps [25,41] or for every 14 of 20 steps [36].

***Threshold of 32 or* 35 mm *Hg.*** These values in millimeters of mercury correspond to approximately 4.3 and 4.7 kPa of pressure, respectively, and relate to capillary arterial pressure (capillary perfusion pressure) in the plantar region of the foot. This is a threshold used as an indirect measure to assess the increase in plantar pressure caused by supporting a person's body weight in their daily activities. The use of this threshold is supported by the fact that even very low external plantar pressures occlude perfusion of the capillary bed into the soft tissues of the foot, significantly impairing the neurovascular response of patients with diabetic foot ulcers compared to patients without ulcers [32,42]. Ferber et al. [42] used a threshold of 32 mm Hg to test and compare two insole-based pressure measurement systems, one that detects vertical plantar pressure (Pedar-X) and the other capillary perfusion pressure (SurroSense RX) and are intended to support the management and treatment of pressure ulcers. In this study, they detected plantar pressure exceeding capillary perfusion pressure (>32 mm Hg), considering this threshold as the minimum pressure that over a 15-min period can lead to minor superficial skin damage [42]. However, in a more recent study, with the same measurement system (SurroSense RX), Abbott et al. [32] found that during 15 min of measurement if 95 % or more of the readings are ≥35 mm Hg capillary perfusion pressure, there is effectively a strong likelihood of ulceration. When these conditions are present an audiovisual and vibratory alert is issued for the patient to correct their gait or posture [32].

***Stratified threshold matrix.*** This concept is suggested by Giacomozzi et al. [43]. It consists of a matrix that assigns a certain stratified threshold based on the patient's risk level, shoe size and foot region. The thresholds were based on reference pressures capable of minimizing the risk of ulceration through measurements inside the shoe every 20 steps per foot [43]. The risk level was previously assigned using the protocol proposed by Uccioli et al. [55], which follows the following classification: 0, low risk - no neuropathy, no history of ulceration or foot deformity; 1, medium risk - with neuropathy, but no history of ulceration or foot deformation; 2, high risk - with neuropathy and foot deformation, but no history of ulceration; 3, very high risk - with neuropathy, foot deformation and history of ulceration [55]. Table 3 shows the respective matrix with proposed thresholds.

### 3.5 Barefoot pressure thresholds

The proposed thresholds are generally indicated for pressures originating inside the shoe. However, and although this approach is the most realistic [49], as it translates the daily life of any patient, four studies have been identified that indicate a pressure threshold with the barefoot [30,[52], [53], [54]]. Barefoot pressure assessment is performed only in a clinical setting and using a pressure platform. This assessment can serve to select regions of interest [53,54] and to classify eventual patients into risk groups [30,52]. As previously described, in Fig. 4 it is possible to consult the distribution between the thresholds applied in the management of plantar pressure and the number of studies that used them, both for applications inside shoes and with the barefoot.

***Threshold of* 700 kPa*.*** An older study by Armstrong et al. [30], revealed that pressures greater than 700 kPa for regions of the plantar forefoot have specificity and sensitivity for screening and identifying groups at higher risk of ulceration. Measurements were performed for 3 steps per foot [30]. More recently, Searle et al. [52], in evaluating the effects of equinus foot condition on plantar pressure in diabetic patients, used the 700 kPa threshold to identify groups with current or recently healed ulcers. This threshold was applied for every 12 steps per foot [52].

***Threshold of* 450 kPa*.*** Telfer et al. [53] and Owings et *al*. [54] used a threshold of 450 kPa pressure to select the regions of interest that exceeded this threshold. The threshold was applied for the first step [54] or for every 12 steps per foot [53].

### 3.6 Summary of study results

The use of objective thresholds to reduce plantar pressure to lower values has revealed in some studies an apparent relationship between pressure reduction and a minimization of ulceration risk [25,32,37,41,[43], [44], [45], [46], [47], [48], [49]]. However, clear evidence of the effectiveness of these pressure thresholds in footwear was more pronounced in four studies [32,39,40,49].

Within footwear, pressure reduction to a threshold of 200 kPa has been achieved by using custom therapeutic footwear, custom insoles, or by modifying their structures to accentuate the reduction in a particular region of interest. For example, Arts et al. [45], using customized footwear, found effective reduction in 61 % of all feet with deformity and specifically in 81 % of feet with midfoot deformity, 44 % of feet with forefoot deformity, and in 62 % of anterior ulcer sites. Using insoles optimized by numerical simulation, Telfer et al. [53] obtained lower plantar pressure peaks in 88 % of the regions of interest compared to devices based on a mold. Generically all shoe modifications significantly reduced the peak pressure at the target locations compared to pre-modification levels [33,37,45,47,48,53]. In particular cases, the metatarsal heads were the most targeted regions in reducing pressure [37].

According to the review conducted, Owings et al. [49] was the precursor of the 200 kPa threshold. In their study, which aimed to measure plantar pressures at the anterior ulcer site and provide information for a given threshold for ulceration prevention, they evaluated 49 participants who wore a Pedar pressure-measuring insole inside extra-deep shoes with custom insoles. The average peak pressure in the shoe at this location averaged 207 kPa while walking for 30 steps. This value was rounded to 200 kPa to provide a target average peak pressure in the shoe [49]. Using this same threshold, Waaijman et al. [39] in a study of 171 participants with diabetes, recently healed ulcer and neuropathy, wearing customized shoes for more than 80 % of the hours of the day reduced the pressure below the 200 kPa threshold, thus leading to a decreased risk of ulcer recurrence [39].

For the threshold of 200 kPa or a reduction by 25 % of the baseline pressure, Bus et al. [40] showed evidence for the 200 kPa threshold using customized shoes. For a participant group of 171 people (apparently the same group used by Waaijman et al. [39]), they were able to decrease ulceration recurrence by 46 % after an evaluation over 18 months. However, the results for the 25 % threshold of reduction in base pressure in the footwear proved to be non-significant in decreasing the relative risk of ulcer recurrence, with the exception that if there is effective adherence to wearing the prescribed footwear for 80 % or more of all steps during the wearer's daily locomotion [40]. Other studies that used this 25 % pressure reduction threshold, as the main objective was to evaluate the ability to optimize and customize shoes, considering the number of modifications required to achieve this end, did not evaluate the effect on ulceration [[46], [47], [48]].

The 40–80 % reduction in initial shoe pressure was based on strategies of gait pattern adaptation through biofeedback prompts and recommendations. Effectively, the participants in these studies were able to correct their posture while walking and thereby reduce the pressures in the places identified as having the highest pressures and classified at risk by the measuring system. Van et al. [36] found that 50 % of the steps for all participants in their study were below the proposed maximum pressure threshold [36], however, these studies did not assess ulcer recurrence rates [25,36,41].

On the proposed threshold for a maximum capillary perfusion pressure (as an indirect measure of plantar pressure threshold) of 32 or 35 mm Hg, and further on the strategy of gait pattern correction by biofeedback prompts, the study by Abbott et al. [32] resulted in a 71 % reduction in the risk of relapse in the intervention group compared to the control group, suggesting that a pressure greater than 35 mm Hg in the shoe for 95 % of the previous 15 min increases the risk of ulceration [32]. Whereas for the 32 mm Hg threshold, Ferber et al. [42] when comparing two measurement systems in insole format, SurroSense *vs.* Pedar-X, only found good to very good correlations for six of the eight SurroSense sensors and a poor correlation in two sensors, analyzing the number of sites that recorded above 32 mm Hg, without assessing their effect on ulceration [42].

The thresholds indicated in the matrix suggested by Giacomozzi et al. [43], for the rearfoot, midfoot, forefoot and toes, also considering the risk level (according to the classification model of Uccioli et al. [55]) of the patient and the size of the shoe worn, have not yet been evaluated in relation to ulceration rates. However, it should be emphasized that after 4 months of wearing the prescribed footwear with application of the proposed thresholds, none of the patients developed plantar ulcers or signs of plantar tissue damage. This protocol may have general validity, however, its application can only be done reliably if the test is performed with the same calibrated measurement system (Pedar) and with the insoles of sizes 36–45 [43].

In the studies where the 450 and 700 kPa barefoot thresholds were used, they showed ability to identify regions of interest and classify risk groups. For the 450 kPa threshold it was possible to identify regions of interest where this value was exceeded [53,54]. At the 700 kPa threshold, it was possible to classify risk groups [30,52]. For example, Searle et al. [52] when evaluating adult diabetic participants with the equinus foot condition, found that they had a significantly higher peak pressure with barefoot than the control group without equinus foot [52] and, Armstrong et al. [30] verified with clear evidence that the peak plantar pressure was significantly higher for patients with ulcers compared to the control group [30].

## 4 Discussion

The studies included in this review presented different objectives and different applications for adopting a certain plantar pressure threshold. Some have focused on outcomes research to prove the effectiveness of reducing pressure by changing the design of custom shoes and insoles. Others were concerned with evaluating whether reducing pressure beyond a certain threshold would have a positive impact on the appearance or treatment of pressure ulcers. Despite the disparate objectives, a common objective was to consider a threshold that was favorable and that would reduce pressure to a safe level for the integrity of the foot. Even so, some studies have revealed objective data on recommended thresholds for plantar pressure, some of which are based on the prevention of ulceration and its recurrence, both for the barefoot (although fewer) and inside the shoe. However, it is reinforced that it is more realistic to measure and monitor plantar pressure inside the shoe [49], as it reflects the daily life of any patient and is an environment conducive to greater pressure and injuries caused by the additional pressure imposed by the shoe.

As for the systems for measuring pressure inside the footwear, due to the high heterogeneity of the systems available on the market, each with its own characteristics (number of sensors, measuring range, sampling rate, etc.), it makes it difficult to select which system is the best and, above all, to make comparisons of the results obtained with this measurement systems [56].

In the studies included in this review, there was considerable variation in the number of regions of interest that the authors considered for analysis, as well as in the number of steps over which the 200 kPa threshold is evaluated. In the literature there are recommendations of a minimum number of 12 steps per foot required to obtain statistically reliable data [57]. It is also important to emphasize the importance of other variables that may increase the risk of injury and influence the pressure inside the shoe. The temperature and humidity inside the shoe can be factors that increase the risk of ulceration [58,59].

The threshold of 200 kPa is the most widely agreed upon among studies, and is the threshold proposed by international guidelines for diabetic foot care, when pressure measurements are taken with a sensor of 2 cm^2^ [3]. However, more evidence is needed to confirm this value and whether dynamic pressure peaks contribute more to ulceration than low sustained pressures within the shoes. The low static pressures in the foot [32], when the patient with diabetic foot adopts a prolonged (1–2 h) and certain posture, may be inducing small irreversible lesions in the foot tissues, such as injuries caused by bedsores in bedridden patients [60].

The heterogeneity of the methodologies adopted by the studies and their broad objectives were also an obstacle to carrying out a meta-analysis. On the other hand, a high number of studies with fair quality, which may present some potential risk of bias, do not allow us to state with evidence about which pressure thresholds are most effective in preventing ulceration, despite the International Working Group on Diabetic Foot [3] admit that the 200 kPa pressure threshold is the most recommended to prevent and accelerate the treatment of possible diabetic foot ulcers. From our perspective, the threshold matrix proposed by Giacomozzi et al. [43] could be a sensible and more easily customizable approach for each patient. This matrix considers the level of risk of the disease (presence or not of neuropathy, ulcers, and anatomical deformities in the foot), the size of the shoe/foot and four regions of interest on the foot, proposing a specific threshold for each situation. However, this approach still needs additional studies to clarify its evidence. On the other hand, we must also highlight those important studies, mainly RCTs and case-control classified with good quality, addressed the proposal of thresholds for 200 kPa or a 25 % pressure reduction.

## 5 Strengths and limitations

A strength is the comprehensive systematic review of the literature using a rigorous methodology according to the PRISMA protocol. This allowed us to have a broader view of the pressure thresholds most used to promote an adequate plantar pressure reduction in diabetic foot. Limitations include the small number of electronic databases that were screened, Scopus, PubMed, and Web of Science. This fact may have excluded some articles, although the selected databases are highly recognized by the scientific community and have a wide multidisciplinary spectrum. An additional limitation was the obstacle created to carrying out a meta-analysis due to the heterogeneity of the methodologies adopted in the studies and the type of applications that implied different objectives in the adoption of pressure thresholds.

## 6 Conclusion

In this systematic review, seven thresholds were identified that are used for the purpose of reducing the risk of diabetic foot ulceration or identifying regions of clinical interest. Five of which are intended for the inside of the shoe: a threshold of average peak pressure of 200 kPa; 25 % and 40–80 % reduction from initial baseline pressure; 32–35 mm Hg for capillary perfusion pressure (as an indirect measure of plantar pressure threshold); and a matrix of thresholds based on patient risk, shoe size and foot region. Two others are intended for the barefoot, 450 and 750 kPa. While measurement inside the shoe is more pertinent and realistic, the thresholds for the barefoot can be useful for identifying regions of interest and classifying the patient into a given level of risk.

The threshold of 200 kPa of pressure inside the shoe is the most consensual among the studies. About the prevention of ulceration or its recurrence, the effectiveness of the proposed threshold matrix and the threshold of reducing baseline pressure by 40–80 % has not yet been evaluated, and the evidence for the remaining thresholds needs further study. Further tests should be considered to prove the effect on ulceration of low static pressures on the foot when prolonged over time compared with dynamic pressures.

Despite some heterogeneity in the methodologies adopted, especially about the measurement systems used, the number of regions of interest to be studied, and the number of steps to be considered for threshold application, this review reveals the path to follow to obtain an objective threshold that is indicative of an effective threshold for diabetic foot ulcer prevention. Furthermore, it is important to highlight the need for a next stage in research, which could focus on additional refinements, such as determining specific thresholds based on the most critical locations in the plantar region while considering foot anatomy variations (such as size, type, deformities, among others). This additional step could be an important contribution to a more precise approach to preventing diabetic foot ulcers based on a certain threshold.

## What is already known

• Plantar pressure monitoring is used to minimize the risk of diabetic foot ulceration and customize footwear suitable for diabetic people, as well as to assist nurses in the management of this condition;
• In practice, a specific plantar pressure threshold is not yet used by the medical community to prevent ulceration and its recurrence, although there are many clinical studies indicating favorable thresholds in certain situations.

## What this paper adds

• It is important to add the information available in the literature to make known the most relevant measurable pressure thresholds to identify the risk of plantar ulceration in diabetic people;
• We provided a systematic review of the literature which reveals the most promising and objective plantar pressure thresholds to be applied in the prevention of diabetic foot ulceration;
• The healthcare professional, physician or nurse, can find a suitable threshold for their diabetic patients or clinical case studies for pressures originating from inside the shoe or with the barefoot.

## Funding sources

This work was developed under a PhD grant (UI/10.13039/100017412BD/151285/2021) awarded to PCM and funded by the Portuguese Foundation for Science and Technology (10.13039/501100001871FCT, Portugal). It also had the support of the “Smart-Health-4-All – Smart medical technologies for better health and care” project (POCI-01-0247-FEDER-046115; LISBOA-01-0247-FEDER-046115), which was co-financed by Portugal 2020, under the Operational Program for Competitiveness and Internationalization (10.13039/501100011929COMPETE 2020) through the European Regional Development Fund (10.13039/501100008530ERDF).

## Data availability statement

This work is a systematic literature review based on searches in the previously mentioned electronic databases, therefore, the data supporting this study were not stored in any other repository. All additional data and information are available upon request from the corresponding author.

## CRediT authorship contribution statement

**Pedro Castro-Martins:** Writing – review & editing, Writing – original draft, Validation, Methodology, Investigation, Formal analysis, Data curation, Conceptualization. **Arcelina Marques:** Writing – review & editing, Supervision, Methodology, Conceptualization. **Luís Coelho:** Writing – review & editing, Methodology, Conceptualization. **Mário Vaz:** Validation, Supervision, Conceptualization. **José Torres Costa:** Validation, Supervision, Conceptualization.

## Declaration of competing interest

The authors declare that they have no known competing financial interests or personal relationships that could have appeared to influence the work reported in this paper.
